# Performance of a commercial multi-sensor wearable (Fitbit Charge HR) in measuring physical activity and sleep in healthy children

**DOI:** 10.1371/journal.pone.0237719

**Published:** 2020-09-04

**Authors:** Job G. Godino, David Wing, Massimiliano de Zambotti, Fiona C. Baker, Kara Bagot, Sarah Inkelis, Carina Pautz, Michael Higgins, Jeanne Nichols, Ty Brumback, Guillaume Chevance, Ian M. Colrain, Kevin Patrick, Susan F. Tapert

**Affiliations:** 1 Exercise and Physical Activity Resource Center, University of California, San Diego, La Jolla, California, United States of America; 2 Center for Wireless and Population Health Systems, University of California, San Diego, La Jolla, California, United States of America; 3 Center for Health Sciences, SRI International, Menlo Park, California, United States of America; 4 Department of Psychiatry, University of California, San Diego, La Jolla, California, United States of America; 5 Department of Psychological Science, Northern Kentucky University, Highland Heights, Kentucky, United States of America; Associazione OASI Maria SS, ITALY

## Abstract

**Purpose:**

This study sought to assess the performance of the Fitbit Charge HR, a consumer-level multi-sensor activity tracker, to measure physical activity and sleep in children.

**Methods:**

59 healthy boys and girls aged 9–11 years old wore a Fitbit Charge HR, and accuracy of physical activity measures were evaluated relative to research-grade measures taken during a combination of 14 standardized laboratory- and field-based assessments of sitting, stationary cycling, treadmill walking or jogging, stair walking, outdoor walking, and agility drills. Accuracy of sleep measures were evaluated relative to polysomnography (PSG) in 26 boys and girls during an at-home unattended PSG overnight recording. The primary analyses included assessment of the agreement (biases) between measures using the Bland-Altman method, and epoch-by-epoch (EBE) analyses on a minute-by-minute basis.

**Results:**

Fitbit Charge HR underestimated steps (~11.8 steps per minute), heart rate (~3.58 bpm), and metabolic equivalents (~0.55 METs per minute) and overestimated energy expenditure (~0.34 kcal per minute) relative to research-grade measures (p< 0.05). The device showed an overall accuracy of 84.8% for classifying moderate and vigorous physical activity (MVPA) and sedentary and light physical activity (SLPA) (sensitivity MVPA: 85.4%; specificity SLPA: 83.1%). Mean estimates of bias for measuring total sleep time, wake after sleep onset, and heart rate during sleep were 14 min, 9 min, and 1.06 bpm, respectively, with 95.8% sensitivity in classifying sleep and 56.3% specificity in classifying wake epochs.

**Conclusions:**

Fitbit Charge HR had adequate sensitivity in classifying moderate and vigorous intensity physical activity and sleep, but had limitations in detecting wake, and was more accurate in detecting heart rate during sleep than during exercise, in healthy children. Further research is needed to understand potential challenges and limitations of these consumer devices.

## Introduction

It is widely recognized that physical activity and sleep are important determinants of health. [1,2] However, the observed associations of physical activity and sleep vary considerably with a variety of health outcomes. [3,4] Although some of this heterogeneity may result from true biological mechanisms, the challenge of precisely measuring these complex habitual behaviors is likely a major contributor. To determine temporal trends and further characterize dose-response relationships with various health outcomes, objective and valid measures of physical activity and sleep are necessary. [5,6]

Tools for the objective assessment of the frequency, intensity (or quality), and duration of physical activity and sleep in adults and children have largely been developed for short-term use (i.e., several days or weeks) within a research and clinical environment (see devices from ActiGraph, GENEActiv, Philips Respironics, etc.). [7,8] However, recent advances in microtechnology, data processing, wireless communication, and battery capacity have resulted in the proliferation of low-cost, non-invasive, devices within the consumer space. These devices have appealing designs and can easily and passively be used by consumers to track their physical activity and sleep over long periods of time, generating an unprecedented amount of data. [9] In 2017, ~102.4 million such devices were shipped worldwide, and this number is predicted to grow to 237.5 million by 2021. [10] Most devices contain Bluetooth connectivity that allows users to automatically transmit data at varying resolutions to the cloud via mobile, web, or computer applications, with minimal or no effort. Corresponding application programming interfaces (API) and standardized OAuth procedures (i.e., a common, industry-standard protocol that enables data access delegation between applications and websites) enable researchers to gain access to the data stored by manufacturers.

Most of the current commercial wearables have multi-sensor capabilities, which has led to refinements in data integration for outcome processing and new opportunities to obtaining data for multiple bio-domains within a single device. For example, the latest generation of consumer-level activity trackers typically use triaxial accelerometry to measure movement (i.e., gravitational acceleration in the anterior-posterior [x], cranial-caudal [y], and medial-lateral [z] planes) and photoplethysmography to measure heart rate (i.e., number of beats per minute) and its variability. Other sensors are of increasingly used to measure a broad range of other bio-signals (e.g., skin temperature) as well as environmental data (e.g., light exposure). Importantly, a combined sensing approach may theoretically address many of the limitations of using one channel of information (e.g., either accelerometry or photoplethysmography alone). [11,12] For example, heart rate monitors can accurately assess high intensity physical activity that accelerometers measure poorly (e.g., cycling on a stationary bicycle), whereas accelerometers can accurately assess low intensity physical activity that heart rate monitors measure poorly (e.g., slow walking). [13] The combination of these data streams through branched equation modeling and/or machine learned algorithms may result in more accurate measurement of physical activity and sleep, [13,14] and allows for the assessment of newly developed metrics such as cardiorespiratory fitness, resting heart rate, heart rate variability, and sleep stages. [15–17]

The easy-to-use nature of these devices led to their proliferation, with a growing use of these commercial devices in health research. Despite their potential advantages, namely the capability of targeting and collecting objective multidimensional data from millions of individuals over time, several challenges and limitation should be addressed. For example, the proprietary nature of the algorithms presents some challenges in data assessment and interpretation. Thus, we believe that within the current unregulated space for consumer wearable technology, it is crucial to evaluate the capability and performance of these devices.

Independent evaluation of the accuracy of the metrics from consumer-level activity monitors has increased recently. [18] However, our understanding of the performance of these devices is still largely unknown. Whereas the vast majority of devices studied to date have relied on accelerometry alone, one combined sensing device that has received substantial scrutiny is the Fitbit Charge HR. Fitbit Charge HR is the first device of the Fitbit family that has multi-sensor capability; released in 2015, it is still used in its original (Charge HR) and updated versions (Charge 2, Charge 3) in clinical and basic research studies. More than 40 studies have independently assessed the validity of one or more metrics from the Fitbit Charge HR in young and middle-aged adults. [19] The methodological rigor of these studies has varied greatly in the use of both laboratory- and field-based assessments, choice of ground truth or “gold standard” measures, and recruitment of a meaningful sample size. Despite these limitations, a growing number of studies suggested that the Fitbit Charge HR provides acceptably valid measurement across a variety of metrics. [15,20–23] However, few studies have been conducted among children and/or adolescents. One study investigated physical activity measures relative to accelerometers in children 10–18 years old with congenital heart disease, [24] another investigated sleep relative to lab-based polysomnography (PSG) in healthy adolescents older than 12 years old, [15] and another investigated energy expenditure relative to indirect calorimetry in healthy adolescents older than 13 years old. [25] The results from these studies are mixed, yet with increasing concern about the importance of physical activity and sleep in youth, validated, cost-effective and appealing instruments are needed for studies of youth health.

In the present study, we assessed the Fitbit Charge HR’s accuracy to measure steps, heart rate, energy expenditure, physical activity intensity, total sleep time, and wake after sleep onset relative to research-grade measures in healthy boys and girls aged 9–11 years old through a combination of laboratory and field tests. We assessed the Fitbit Charge HR performance for measuring sleep in a subset of boys and girls during an unattended overnight at-home PSG assessment.

## Materials and methods

### Participants

Potential participants were recruited from the San Diego Unified School District and the greater San Diego area via in-class announcements and email listservs. Eligible participants were 9–11 years old; able to understand English or Spanish; do light to moderate physical activity for 60 minutes; and walk, jog, or run unassisted. Potential participants were excluded if they had any functional limitation or medical condition prohibiting their ability to be physically active or used medications to alter body weight or metabolism. All study procedures were approved by the University of California, San Diego Human Research Protections Program. All participants provided written assent, with written consent to participate from their parent or legal guardian. Participants were provided monetary compensation for their participation.

### Procedures and measures

Participants completed a 2- to 3-hour testing session at the Exercise and Physical Activity Resource Center at University of California, San Diego. Prior to the start of testing, participants were asked to self-report their age, sex, and handedness. Weight (to the nearest 0.1kg) and height (to the nearest 0.1cm) were measured using a calibrated digital scale and stadiometer (Seca 703, Seca GmbH & Co. KG.). Participants were then fitted with a Fitbit Charge HR on their non-dominant wrist. The Fitbit Charge HR is not considered to be a medical device. It is made of a flexible, durable elastomer material similar to that used in many sports watches, with a surgical-grade stainless steel buckle. It is lightweight, 0.83 cm wide, and fits wrists that are 5.4 to 8.7 inches in diameter. The device contains a triaxial accelerometer, optical heart rate monitor, altimeter, and vibration motor. Precise performance specifications of each sensor are not made available by the manufacturer. The devices used in this study were initialized using generic, study-specific Fitbit accounts and non-identifiable participant information (i.e., sex, height, weight, and handedness). At the time of testing, Fitbit accounts could not be established with a user age less than 13 years old, so all accounts were established using the age of 13. Data from the generic, study-specific Fitbit accounts were downloaded via Fitabase (Small Steps Labs LLC, San Diego, CA), a third-party research platform designed to collect Fitbit data at granularity useful to researchers (i.e., 1-minute epochs). The measures from the Fitbit Charge HR examined in the present study include heart rate, caloric energy expenditure, steps, physical activity intensity, and sleep.

Participants were also fitted with a portable three-lead electrocardiogram (ECG) used to measure heart rate in beats per minute (bpm; Biopac Systems, Inc., Goleta, CA); a portable indirect calorimeter used to measure caloric energy expenditure in kilocalories (kcal) and physical activity intensity in metabolic equivalents of task (METs; Cosmed K4B2, Cosmed Inc., Rome, Italy); and a person-worn video camera mounted on a harness such that it consistently pointed at the participant’s feet and lower legs so that steps could be counted (GoPro Hero, GoPro, Inc., San Mateo, CA). All devices were fitted according to manufacturer recommendations, and recording start times were synchronized with the Fitbit Charge HR. The devices collected data throughout the testing session, which consisted of 14 structured tasks grouped into domains of sitting, stationary cycling, treadmill walking or jogging, stair walking, outdoor walking, and agility drills (Table 1). The tasks were selected to represent the types of physical activity and exercise that children the age of interest might do on a regular basis.

**Table 1:** Laboratory and field tests conducted for comparison of the Fitbit Charge HR and research-grade measures.

Category	#	Activity	Time	Description
**Sitting**	1	Quiet	5 minutes	Sitting quietly
	2	Music	5 minutes	Listening to music
	3	Game	5 minutes	Playing iPad game
		**Effort**	**Time**	**Description**
**Stationary Cycling**	4	Moderate (0.8W/kg)	6 minutes	Cycling @ 55+ rpm
	5	Vigorous (1.2W/kg)	6 minutes	Cycling @ 55+ rpm
		**Speed**	**Time**	**Description**
**Treadmill Walking or Jogging**	6	3 mph	6 minutes	Walking on a treadmill
	7	4 mph	6 minutes	Running/fast walking on a treadmill
	8	3 mph with backpack	6 minutes	Walking on a treadmill with backpack weighing 10% of body weight
		**Direction**	**Flights**	**Description**
**Stair Walking**	9	Up	5	Going up stairs
	10	Down	5	Going down stairs
		**Course**	**Length**	**Description**
**Outdoor Walking**	11	Uphill	200m	Walking up a marked 200m uphill
	12	Flat	400m	Walking a marked 400m flat course
	13	Downhill	200m	Walking down a marked 200m downhill
		**Course**	**Time**	**Description**
**Agility Drills**	14	Ladder Drills	5 minutes	Agility Ladder Drills
		Flag/Cones Drills		Shuttle runs and agility drills

Data from the aforementioned devices were extracted by trained research staff and metrics were aligned to the granularity of the corresponding Fitbit Charge HR metrics (i.e., minute-level values). BPM from the ECG and METs from the indirect calorimetry were aggregated to the minute-level after the removal of aphysiologic data (i.e., 1-second heart rates <50 bpm or >220 bpm and 15-second METs <1 or >12). Physical activity intensity from indirect calorimetry was defined as sedentary <1.50 METs, light 1.50–2.99 METs, moderate 3.00 to 5.99 METs, and vigorous as ≥6.00 METs. [26] To determine step count, two trained research staff members independently viewed videos of the testing sessions and counted the number of steps a participant took and counts were averaged. For cases in which step counts varied by more than 3%, a third trained research staff member counted and the values from the two closest counts were averaged.

Upon completion of the testing session, participants were sequentially asked if they would be willing to wear the Fitbit Charge HR at home during an unattended overnight polysomnographic (PSG) assessment of their sleep performed according to the American Academy of Sleep Medicine (AASM) guidelines. [27] Those who agreed had a trained technician arrive at their home approximately one hour before their usual bedtime to prepare for the recording. They were then fitted with a portable PSG system that included electroencephalography (EEG; 2 leads: C3/4 referenced to the contralateral mastoids; sample rate 256 Hz with a band-pass filter of 0.3-35Hz), submental electromyography (EMG), bilateral electrooculography (EOG), and ECG (Compumedics Somte´ PSG; Compumedics). Additional standard sensors were used to record breathing pattern, blood oxygen levels, and leg movements to confirm that participants did not suffer from sleep disorder(s). Participants (or their parents) selected lights-out and lights-on times. To synchronize the Fitbit Charge HR with the PSG system, “sleep mode” was initiated by pressing and holding the Fitbit Charge HR event-marker button for more than 2 seconds when the technician manually started the PSG recording in the evening, and again upon completion in the morning. The technician returned in the morning after the assessment to recover the equipment and data.

Wake and sleep (N1, N2, N3 and REM) from PSG were scored by a trained sleep laboratory technician in 30-second epochs according to AASM guidelines. [27] Standard sleep parameters were calculated: Time in bed (TIB), defined as the period from self-reported lights-out until PSG-defined final awakening; sleep onset latency (SOL), defined as the time from self-reported lights-out until the first epoch of any sleep stage; total sleep time (TST) in minutes, defined as all epochs of sleep during TIB; sleep efficiency (SE), as TST/TIB; wake after sleep onset (WASO) in minutes, defined as all epochs of wake during the TIB period; time spent in each stage of sleep (N1, N2, N3 and REM) in minutes.

PSG 30-second epochs were aggregated to the minute-level as follows: two consecutive PSG 30-second epochs scored as N1, N2, N3 or REM, were coded as sleep. If one or both of the consecutive PSG 30-second epochs were scored as wake, that minute was coded as wake. [28,29] The corresponding minute-level metrics from the Fitbit Charge HR were defined as ‘sleep’, ‘restless’, and ‘wake’. ‘Restless’ and ‘wake’ were combined and treated as wake, thus enabling the calculation of TST, and WASO. PSG-device comparisons were performed for TST, SOL and WASO.

### Sample size and analyses

Sample sizes in validity studies vary greatly. In the present study, the primary aim was to determine the agreement between measures. Thus, we aimed to include approximately 60 participants in the physical activity and exercise portion of the study, which would result in 95% confidence intervals around the limits of agreement equal to 0.44 standard deviations of the differences between measurements. Due to time and cost constraints, a subset of 26 participants from the physical activity sample completed the sleep portion of the study. This is a similar size to that used in other validity studies that included PSG. [15,30,31]

The agreement between measures from the Fitbit Charge HR and corresponding research-grade measures were examined using the Bland-Altman method. The mean of differences for each of the measures being compared (i.e., the average ‘bias’) were calculated along with 95% confidence intervals calculated as the mean difference ±1.96 times the standard error of the differences. Positive bias indicated that the Fitbit Charge HR underestimated the metric of interest compared to research-grade tools, whereas negative bias indicated that the Fitbit Charge HR overestimated these metrics. The limits of agreement were defined as the mean of the differences ±1.96 times the standard deviation of the differences. A Bland-Altman plot of the mean of the differences by the means of the measures, along with the limits of agreement, was used for visual judgment of how well the methods of measurement agree. Mean absolute percentage error (MAPE) was calculated as the average of absolute differences between the measures, divided by the relevant research-grade measure, multiplied by 100. Accuracy and corresponding Cohen’s kappa, sensitivity, and specificity were calculated for categorical values of physical activity intensity (i.e., sensitivity: ability of the device to correctly classify moderate and vigorous activities; specificity: ability of the device to correctly classify sedentary and light activities) and sleep states (i.e., sensitivity: ability of the device to correctly classify PSG sleep epochs; specificity: ability of the device to correctly classify PSG wake epochs). Assumptions of the normality of underlying raw data were evaluated by inspection of Q-Q plots of residuals, and they were found to be normally distributed. Additionally, paired-sample t-tests were calculated to assess if differences in measures were statistically significant (p-value less than 0.05) and linear regression was used to explore the association between the amount of bias and the magnitude of measurement.

## Results

### Participant characteristics

A convenience sample of 60 participants was recruited for participation in the study. No participant assessed for eligibility was excluded. However, after 1 participant completed the testing, there was an error in syncing their Fitbit that resulted in minute-level data being unavailable. The exact source of the error is unknown, but it was likely due to incorrect initialization of the Fitbit. The participant was excluded from all analyses. Additional exclusions were task specific and reasons included participant’s legs being too short to comfortably reach the pedals of the cycle ergometer, ECG electrodes falling off, the facemask of indirect calorimeter becoming loose, or unusable video footage. Participant numbers by task, as well as descriptive statistics (means and standard deviations) for each of the measures of interest are provided in the supplemental results (see S1 File). The mean (standard deviation [SD]) age of participants was 9.9 (0.7) years, and 52.5% were female. Mean (SD) height was 1.4 (0.1) meters and weight was 36.8 (8.8) kilograms.

### Steps

**Fig 1:**
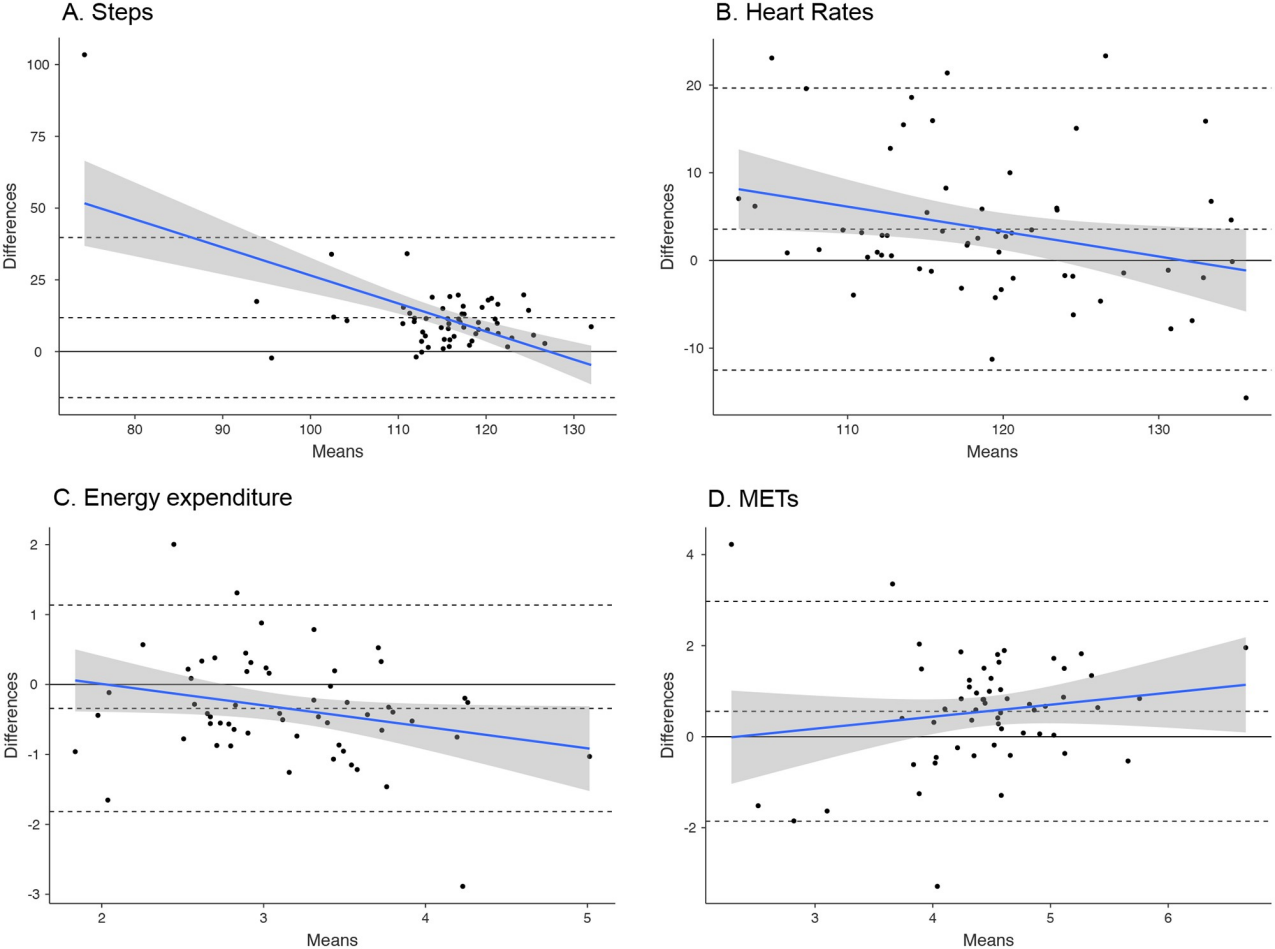
Bland-Altman plots for average steps (A), heart rate (B), energy expenditure (kcal) (C) and METs (D) per minute. The central dotted line represents the Bland-Altman biases; the thin dotted lines represent the Bland-Altman agreement limits.

Excluding those tasks completed while sitting and on the cycle ergometer, the Fitbit Charge HR underestimated step count by a mean of 11.8 steps per minute (95% CI [8.11; 15.59]; p <0.001) compared to direct observation, with 1 participant falling outside the agreement limits (upper: 39.7, lower: -16.1, see Fig 1A). The largest disagreement was during fast (4.0 mph) treadmill walking/jogging (Fitbit underestimated mean: 20.5, upper limit: 60.6, lower limit: -19.6), and the smallest disagreement was while walking up stairs (Fitbit underestimation mean: 3.11, upper limit: 31.37, lower limit: -25.14). The mean absolute percentage error between the two measures was 9.9%. There was a negative association between the amount of bias and mean steps (*β* = - 0.9, p<0.001).

### Heart rate

Throughout all 14 tasks the Fitbit Charge HR underestimated heart rate by a mean of 3.58 beats per minute (95% CI [1.42; 5.74], p < .001) compared to the ECG, with 4 participants falling outside the agreement limits (upper: 19.66, lower: -12.50, see Fig 1B). The largest disagreement was during light cycling (Fitbit underestimated mean: 11.4, upper limit: 43.3, lower limit:-20.6) and the smallest disagreement was during agility skills (Fitbit overestimated mean: -.72, upper limit: 29.56, lower limit: -31.01). The mean absolute percentage error was 3.3%. There was a negative association between the amount of bias and mean heart rate (*β* = -0.3, p < .05).

### Energy expenditure

Throughout all 14 tasks the Fitbit Charge HR overestimated energy expenditure by a mean of 0.34 kcal per minute (95% CI [-.54; .14], p <0.01) compared to the indirect calorimeter, with 3 participants falling outside the agreement limits (upper: 1.14, lower: -1.82, see Fig 1C). The largest overestimation was during ladder drills (mean: -1.88, upper limit: -3.58, lower limit: -0.22) and the smallest disagreement was during music listening (mean: 0.12, upper limit: 0.63, lower limit: -0.39). The mean absolute percentage error between the two measures was 11.4%. There was a negative association between the amount of bias and mean energy expenditure (*β* = -0.3, p = .05).

### METs

Throughout all 14 tasks the Fitbit Charge HR underestimated by a mean of 0.55 METs per minute (95% CI [.23; .88], p<0.01) compared to the indirect calorimeter, with 3 participants falling outside the agreement limits (upper: 2.97, lower: -1.85, see Fig 1D). The largest disagreement was during moderate cycling (Fitbit underestimated mean: 2.84, upper limit: 5.46, lower limit: .21) and the smallest disagreement was during uphill walking (Fitbit underestimated mean: 11.4, upper limit: 43.3, lower limit: -20.6). The mean absolute percentage error between the two measures was 11.8%. There was no association between the amount of bias and mean METs.

### Physical activity intensity

Throughout all 14 tasks the Fitbit Charge HR had an overall accuracy of 84.8% in classifying sedentary or light activity and moderate or vigorous activity (i.e., 2,935 minutes correctly classified out of 3,463 total minutes; Cohen's kappa = 0.65), a sensitivity of 85.4% in identifying moderate or vigorous activity (i.e., 2,084 minutes correctly classified out of 2,439 total minutes of moderate or vigorous activity), and a specificity of 83.1% in identifying sedentary or light activity (i.e., 851 minutes correctly classified out of 1,024 total minutes of sedentary or light activity).

### Sleep/wake pattern and heart rate during sleep

A total of 26 participants (13 female) were invited to participate in the at-home sleep portion of the study in the order in which they completed the physical activity portions of the study. Three participants were excluded from the sleep analysis and 4 from the HR analysis during sleep due to PSG device malfunction (i.e., device did not record and store data properly for an unknown reason), short sleep time (3 standard deviations outside the group mean), or failure in the alignment of the Fitbit Charge HR to PSG, resulting in a final sample of 23 (sleep) and 22 (HR).

**Fig 2:**
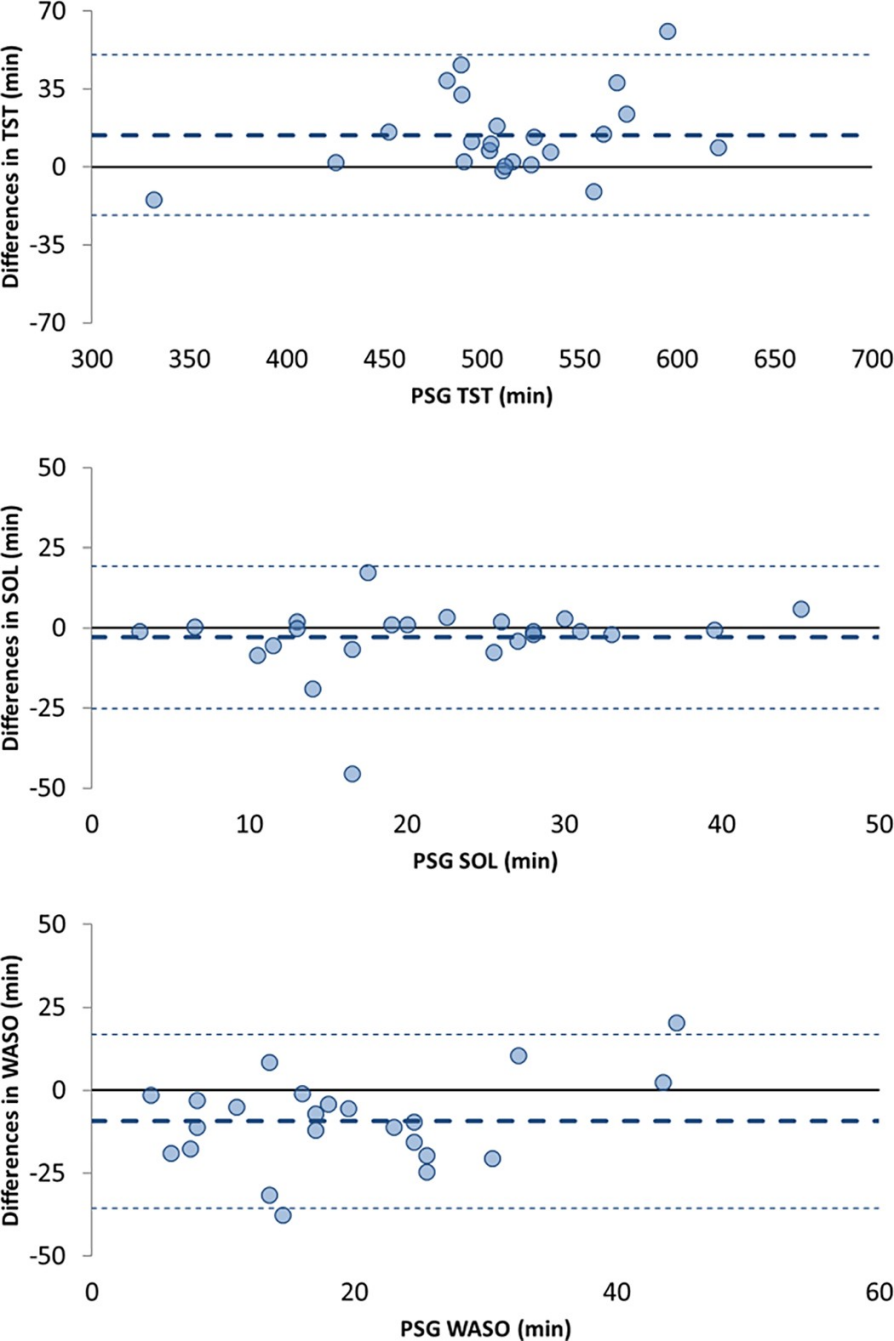
Bland-Altman plots for Total Sleep Time (TST), Sleep Onset Latency (SOL), and Wake After Sleep Onset (WASO). The central dotted lines represent the Bland-Altman biases; the thin dotted lines represent the Bland-Altman agreement limits. *PSG is considered the gold standard for sleep assessment, therefore, mean bias was plotted against the PSG measurement alone and not the average of measures from PSG and the Fitbit Charge HR.

From PSG, participants spent 553±59 (mean ± SD) min in bed, with a SOL of 22 ± 10 min, a WASO of 20 ± 11 min, and sleep efficiency of 93 ± 2%. They had 4 ± 1% of time in N1 sleep, 33 ± 10% in N2 sleep, 42 ± 11% in N3 sleep, and 21 ± 5% in REM sleep. The Fitbit Charge HR underestimated PSG TST (mean ± SD of bias: 14 ± 18 min; p = 0.001) and overestimated PSG WASO (mean ± SD of bias: -9 ± 13 min; p = 0.003). A maximum of 2 participants exceeded the Bland-Altman plot agreement limits for these measures (see Fig 2). There were no associations between the amount of bias and mean PSG TST and WASO. From the epoch-by-epoch analysis at the minute level, the Fitbit Charge HR had an overall accuracy of 92.1% in classifying awaking and sleep (i.e., 11,676 minutes correctly classified out of 12,674 total minutes; Cohen's kappa = 0.53), a sensitivity of 95.7% in identifying sleep (i.e., 11,010 minutes correctly classified out of 11,503 total minutes of sleep), and a specificity of 56.9% in identifying awaking (i.e., 666 minutes correctly classified out of 1,171 total minutes of awaking).

**Fig 3:**
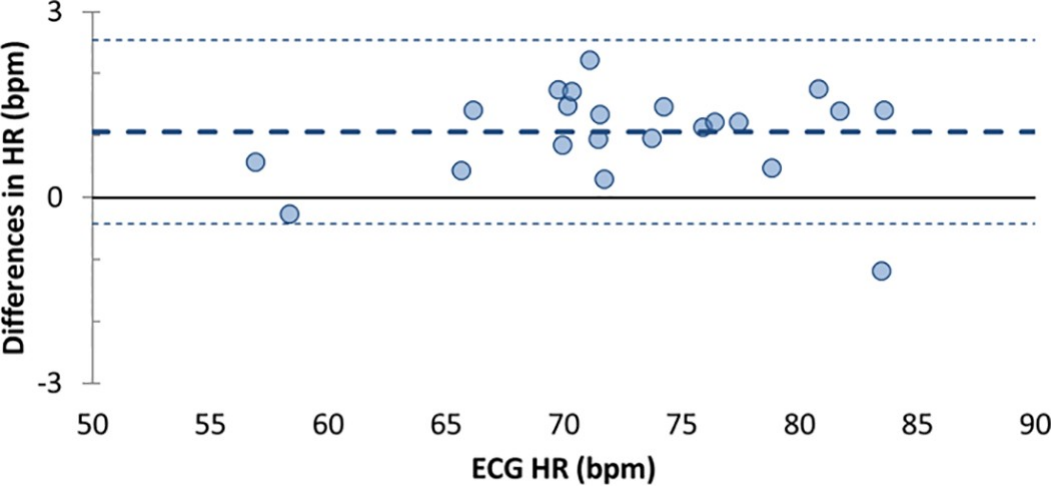
Bland-Altman plots for nocturnal Heart Rate (HR). The thick dotted lines represent the Bland-Altman bias; the thin dotted lines represent the Bland-Altman upper and lower agreement limits.

Mean participant HR from ECG was 72.60 ± 7.07 bpm, and 71.54 ± 7.04 bpm from the Fitbit Charge HR. The Fitbit Charge HR underestimated ECG HR (mean ± SD of bias: 1.06 ± 0.75 bpm; p<0.001), with one participant falling outside of falling outside the agreement limits (upper: 2.55, lower: -0.42, Fig 3).

## Discussion

The use of consumer-level activity trackers in health research is increasing. To date, more than 400 research studies have utilized Fitbit devices. [19] However, the performance of these devices is still largely unknown. The present study provided performance outcomes for the accuracy of Fitbit Charge HR in estimate measures of physical activity and sleep against research-grade evaluation, across different tasks and conditions in a sample of healthy children.

For physical activity, the Fitbit Charge HR underestimated steps, heart rate, and METs, and overestimated energy expenditure, with no more than 4 participants falling outside of the Bland-Altman agreement limits. Estimates of bias varied across tasks, but on average, the mean absolute percentage error was less than 12%. Interestingly, while previous studies did not observe proportional bias in Bland-Altman analyses of energy expenditure among adults, [32] in the present study we observed small but statistically significant associations between each measure and the magnitude of the measure, with the exception of METs. Additional research is needed to determine if this systematic bias exists in other samples of children and if a linear calibration equation might improve the accuracy of these measures.

The measurement of physical activity intensity is particularly important in health research, because the volume of moderate and vigorous activity is a strong predictor of morbidity and mortality at all stages of life. According to national and international guidelines, children and adolescents should accumulate at least 60 or more minutes of moderate to vigorous physical activity daily to experience health benefits, including improved bone and muscle strength, weight control, and psychological well-being. [33] Thus, to accurately quantify adherence to guidelines, valid measurement of moderate and vigorous activity is required. In the present study, the Fitbit Charge HR showed an overall accuracy of 85.4% in distinguishing moderate and vigorous activity from sedentary and light activity, that was similar to research-grade accelerometry. Specifically, when compared to activity intensity determined using similar indirect calorimetry methods to those used in the present study, the commonly used ActiGraph and Actical accelerometers have an accuracy in classifying moderate and vigorous activity among children that ranges from 83% to 86%. [34] It is important to highlight that aforementioned research-grade accelerometers must be hip-worn to measure physical activity intensity, whereas the Fitbit Charge HR is worn on the wrist. Wrist-based physical activity measurement has been shown to be more acceptable to participants than hip-based measurement and allows for the measurement of 24-hour activity, including sleep, as opposed to only daytime activity. [7,35]

The Fitbit Charge HR performance in detecting common sleep metrics, such as TST and WASO, relative to unattended at-home PSG in this group of healthy children showed mean biases of less than 15 min, with no more than 2 participants falling outside the Bland-Altman agreement limits, and with no significant bias for SOL. While the device underestimated HR during the night, the average bias was about 1 bpm, which was similar to the average HR bias (< 1 bpm) found in 12–21 y old healthy adolescents in whom the same device model was tested against standard ECG during sleep. [36] Although in the current study the Fitbit Charge HR performance was only compared to PSG (as opposed to actigraphy), the ability of the Fitbit Charge HR to detect sleep parameters, relative to PSG, was similar to previous reports of research-grade actigraphy performance in children. [37] In that study, authors analyzed in-lab overnight sleep in 115 children and adolescents (3–18 y old). Similar to our results, motion-based actigraphy devices overestimated PSG WASO and underestimated PSG TST in school-age children (6–12 y); however, the devices’ performance depended on several factors including sensitivity threshold, scoring algorithm, developmental age group, and sleep disordered breathing status. [37]

In our study, EBE analysis on a minute-by-minute basis indicated that the Fitbit Charge HR was able to identify sleep and wake epochs with 92% accuracy. The Fitbit Charge HR correctly identified 96% of sleep epochs. However, in concordance with research-grade actigraphy, [38] the Fitbit Charge HR had a lower specificity, accurately detecting wake epochs at a rate of 56%. Validation studies of actigraphy in children have generally reported a similar pattern of high sensitivity and relatively lower specificity (for a review, see [8]). Our findings are also consistent with other validation studies examining the use of Fitbit devices in healthy adolescent [15,39] and adult samples. [16,40] For example, Fitbit Charge HR showed a specificity of 42% in a sample of 12–21 y old healthy adolescents, [36] while a similar Fitbit model (Fitbit Charge 2) showed a specificity of 61% in a sample of 19–61 y old adults. [41]

Importantly, when PSG is not accessible, clinical grade actigraphy is currently considered an accepted alternative for measuring sleep in non-laboratory settings. Actigraphy has been widely adopted in research, and it relies on openly validated standard algorithms. Due to a simple rationale (motion = wake, motionless = sleep), its performance in sleep/wake assessment is largely predictable and characterized by a well-known limitation in the assessment of motionless wake (poor specificity). [29] Despite in its infancy, the new generation of consumer sleep trackers has the ability of differentiating sleep stages (‘light’, ‘deep’, ‘REM’) in addition to tracking sleep and wake. This is possible due to the measure and use of cardiac function data and other features, in addition to motion, showing sleep stage differentiation. [43] This implementation is particularly relevant, allowing for the tracking of sleep composition across periods of maturation such as during childhood and adolescence.

However, despite the theoretical advantages in combining cardiac function data and motion for sleep/wake assessment (particularly in the classification of motionless wake), low specificity seems to still be a limitation of multi-sensor sleep trackers (see for review). [29,42,43] The level of accuracy that we will be able to reach by using peripheral physiology and motion and advancing signal processing, to estimate EEG-based sleep macrostructure, is still unknown. Currently, the performance of standard actigraphy and commercial wearable technology is not dissimilar when both devices are evaluated in the same study and compared against PSG. [43] Considering factors like devices cost, implementation, data accessibility, multi-sensors capability, and multi-systems integration, consumer wearable technology may offer an appealing solution for measuring sleep on a large scale.

While there is increasing study of physical activity and sleep in youth using consumer-level activity trackers, few studies to-date have used these objective measures to examine the links between physical activity, sleep, and other behavioral and neuropsychological indicators of well-being or dysfunction. The Adolescent Brain Cognitive Development (ABCD) study is a multi-site study following over 11,800 9- to 10- year-olds prospectively into young adulthood to increase our understanding of brain development, health, and psychosocial functioning in youth that is utilizing the device validated within the present study. [44] This study provides the opportunity to use a relatively inexpensive, consumer-level activity tracker to examine the diverse biological and environmental mechanisms contributing to physical activity and sleep, as well as the impact of physical activity and sleep on mental and physical health throughout development. The literature to-date demonstrates relationships between poor sleep quality and quantity, and large weekend-weekday differences in sleep and increased substance use among adolescents. [45,46] Insomnia has also been associated with poor mental health outcomes, including depression and suicidal ideation among youth. Low physical activity and high amounts of sedentary activity are associated with poorer physical health, including obesity and related medical disorders such as diabetes mellitus type 2, and psychosocial dysfunction. [47] However, a dearth of prospective studies on these reciprocal relationships makes the potential contribution of ABCD key to understanding the bidirectional relationships between physical activity, sleep, and health across development.

The present study has several important strengths and adds significantly to existing research on this topic. First, we used a relatively large sample of boys and girls who completed a variety of laboratory-, field-, and home-based tasks. Most validation studies of consumer-level activity monitors are restricted to laboratory-based assessments only. [15,20–23] The tasks that participants completed were selected to reflect activities that 9- to 11-year-old children might typically do throughout the day, and they included a variety of ambulatory and non-ambulatory activities across the range of intensity classification from sedentary to vigorous. In addition, we evaluated multiple measures from one device, the Fitbit Charge HR, which was worn as instructed by the manufacturer. Many previous validation studies have attempted to validate multiple devices simultaneously (i.e., multiple devices were worn on the same wrist at the same time), and therefore, they may be inherently flawed due to the fact that devices were not worn according to manufacturers’ specifications. Lastly, our criterion measures for physical activity are commonly used in physical activity and exercise research, and for sleep, we used unattended at-home PSG.

The findings of the present study should be considered within its limitations. First, little to no consensus exists for the ideal combination of laboratory-, field-, and home-based tasks for assessing the validity of an activity tracker. Similarly, consensus is lacking regarding the optimal physical activity measures for criterion measures. Given the lack of gold standard measures for physical activity, it is not possible to truly know the source of bias (e.g., bias in steps could be due to poor human interpretation of what constitutes a step). Further, the Fitbit Charge HR was not designed for use in children, and because the algorithms used to derive the various metrics are proprietary, we are unable to determine how differences in anthropometry and heart physiology influence accuracy. Regarding sleep, although unattended at-home PSG is feasible, technically adequate, and well-tolerated in school-aged children when performed under research conditions, [48] and may be used in wearable validation studies, in-lab PSG is the true gold standard for assessing the performance of sleep-tracking technology. [29,42] Controlled laboratory investigations using gold standard PSG assessment in a larger sample of children, including those with clinical sleep disturbances, are needed, which would also allow testing for potential factors (e.g., sex, age, sleep disruption) that could affect device performance. Also, in wearable validation studies, PSG-derived sleep outputs have been suggested as the ‘true standard’ in Bland-Altman plots. [29,42] However, an ongoing and unsolved issue in consumer wearable technology validation is that AASM visual sleep scoring rules are subjected to human interpretation, challenging the concept of PSG sleep as the true reference for comparison. [29] Given the frequently observed relationship between sleep disruption and consumer sleep-tracking technology performance (greater PSG-device biases with greater amount of PSG wake), [43] our study results can only be extended to healthy children. In addition, a methodological limitation needs to be considered. The procedure adopted for converting 30-s PSG epochs into 1-min epochs, necessary to match the Fitbit 1-min sleep scoring, may have resulted in an overinflating of the amount of PSG wake, potentially impacting the assessment of the Fitbit performance in wake detection. [29] Additionally, the development of consumer-level activity trackers is outpacing the ability to independently validate them, and it remains unknown if these results extend to future iterations of the same device. Lastly, there is variability in the analytic approaches used in validation studies of this type. While our analyses centered on Bland-Altman methods that are often robust even when the distribution of differences is not normal, the normality hypothesis was not formally evaluated.

## Conclusions

Cost-accessible, comfortable activity trackers that provide objective measurements among children are critically important to understanding the influence of physical activity and sleep on health. This is among the first studies to systematically evaluate the validity of a common multi-sensors consumer-level activity tracker to measure physical activity and sleep in children. The results of the present study provide researchers, clinicians, and consumers alike with estimates of accuracy and bias in multiple physical activity and sleep metrics across a variety of tasks. On balance, the ease of use, low cost, and palatability to youth make these multi-sensors consumer devices as promising tools for the study of youth physical activity, sleep, and health. More effort in evaluating challenges and limitation in using this technology is clearly needed.

## Supporting information

S1 File (DOCX)

S1 Data (CSV)

S2 Data (CSV)

S3 Data (CSV)
