# Supplementary material for: Performance of a commercial multi-sensor wearable (Fitbit Charge HR) in measuring physical activity and sleep in healthy children
Source: PLoS One. 2020 Sep 4;15(9):e0237719. doi: 10.1371/journal.pone.0237719 (PMC7473549; doi:10.1371/journal.pone.0237719)
Supplement: S1 File — (DOCX) [file pone.0237719.s001.docx]

Appendix: Participant numbers, as well as descriptive statistics for each metrics (i.e., heart rate, steps, energy expenditure and MET levels) are provided by groups of activities and specific testing conditions (see Table 1 in the manuscript for a description of each activity and corresponding group).

*1. Heart Rate*

|  | Descriptive | | | | Bland-Altman | | | |
| --- | --- | --- | --- | --- | --- | --- | --- | --- |
|  | N | M (SD) BP | M (SD) FB | MAPE | Bias | Lower | Upper | 95% CI |
| Average | 58 | 121 (8.15) | 117 (10.3) | 3.3 | 3.58 | -12.50 | 19.66 | [1.42; 5.74] |
| Group |  |  |  |  |  |  |  |  |
| 1 | 55 | 91.2 (18.7) | 84.7 (10.1) | 6.6 | 6.54 | -29.86 | 42.93 | [1.52; 11.06] |
| 2 | 55 | 125 (15.8) | 116 (19.4) | 7.2 | 8.78 | -20.51 | 38.06 | [4.74; 12.8] |
| 3 | 57 | 132 (8.61) | 132 (11.9) | 0 | .65 | -17.43 | 18.73 | [-1.80; 3.10] |
| 4 | 58 | 137 (11.6) | 128 (17.1) | 6.6 | 9.35 | -29.41 | 48.11 | [4.15; 14.6] |
| 5 | 56 | 123 (10.2) | 124 (12.8) | -.8 | -.98 | -20.48 | 18.53 | [-3.65; 1.69] |
| Activity |  |  |  |  |  |  |  |  |
| 1 | 55 | 91.1 (20.1) | 85 (11.8) | 6.6 | 6.05 | -35.08 | 47.18 | [.38; 11.7] |
| 2 | 55 | 90.3 (18.2) | 83.3 (9.85) | 7.8 | 7.02 | -26.61 | 4064 | [2.38; 11.17] |
| 3 | 55 | 92.2 (18.3) | 85.6 (9.83) | 6.5 | 6.54 | -29.52 | 42.60 | [1.56; 11.15] |
| 4 | 52 | 123 (12) | 111 (17) | 9.8 | 11.4 | -20.6 | 43.3 | [6.82; 15.9] |
| 5 | 50 | 133 (13.1) | 130 (18.4) | 2.3 | 5.97 | -30.68 | 42.62 | [0.66; 11.13] |
| 6 | 55 | 124 (11.5) | 121 (11.7) | 2.4 | 2.35 | -18.59 | 23.28 | [-.54; 5.24] |
| 7 | 55 | 143 (12.4) | 144 (17.1) | -.7 | -1.36 | -40.76 | 38.03 | [-6.80; 4.07] |
| 8 | 54 | 129 (12.8) | 128 (12.5) | .8 | 0.96 | -15.35 | 17.27 | [-1.31; 3.23] |
| 9 | 58 | 143 (14.5) | 135 (25.4) | 5.6 | 8.59 | -50.64 | 67.82 | [.64; 16.5] |
| 10 | 55 | 130 (12.2) | 121 (12.9) | 6.9 | 9.34 | -14.55 | 33.22 | [6.04; 12.63] |
| 11 | 56 | 127 (11.7) | 132 (17.7) | -3.9 | -5.69 | -36.21 | 24.83 | [-9.86; -1.52] |
| 12 | 56 | 123 (10.7) | 122 (14.1) | .8 | .872 | -22.56 | 24.30 | [-2.33; 4.07] |
| 13 | 56 | 119 (15) | 118 (11.2) | .8 | -.72 | -31.01 | 29.56 | [-4.86; 3.41] |
| 14 | 56 | 141 (13.2) | 143 (19.2) | -1.4 | -1.76 | -52.99 | 49.48 | [-8.76; 5.24] |

M (SD) = Mean and standard deviation ; BP = Biopac ; FB = Fitbit ; MAPE = Mean absolute percentage error.

*2. Steps*

|  | Descriptive | | | | Bland-Altman | | | |
| --- | --- | --- | --- | --- | --- | --- | --- | --- |
|  | N | M (SD) P | M (SD) FB | MAPE | Bias | Lower | Upper | 95% CI |
| Average | 58 | 121 (7.24) | 109 (14.1) | 9.9 | 11.8 | -16.1 | 39.7 | [8.11; 15.59] |
| Group |  |  |  |  |  |  |  |  |
| 3 | 57 | 139 (8.21) | 125 (14.5) | 10.1 | 14.9 | -11.5 | 41.3 | [11.3; 18.48] |
| 4 | 58 | 84.4 (9.29) | 74.3 (14.2) | 11.9 | 10.1 | -20.5 | 40.7 | [5.98; 14.2] |
| 5 | 56 | 112 (6.25) | 101 (15.7) | 9.8 | 10.3 | -20.6 | 41.2 | [6.05; 14.5] |
| Activity |  |  |  |  |  |  |  |  |
| 6 | 56 | 135 (9.83) | 122 (12.4) | 9.6 | 13.83 | -5.02 | 32.69 | [11.26; 16.41] |
| 7 | 54 | 156 (12.6) | 135 (23.4) | 13.5 | 20.5 | -19.6 | 60.6 | [14.9; 26.1] |
| 8 | 54 | 130 (7.8) | 119 (19) | 8.5 | 11.6 | -24.8 | 48 | [6.52; 16.7] |
| 9 | 58 | 80.9 (11.8) | 77.8 (15) | 3.7 | 3.11 | -25.14 | 31.37 | [-0.68; 6.91] |
| 10 | 58 | 90.7 (23.2) | 70.9 (19.9) | 22 | 19.8 | -33.6 | 73.1 | [12.6; 26.9] |
| 11 | 56 | 106 (7.64) | 97.8 (17.1) | 7.5 | 8.38 | -22.94 | 39.70 | [4.10; 12.7] |
| 12 | 56 | 118 (8.36) | 107 (16.7) | 9.3 | 11.8 | -22 | 45.6 | [7.19; 16.64] |
| 13 | 56 | 106 (13.2) | 96.5 (18.3) | 8.5 | 9.94 | -24.02 | 43.91 | [5.30; 14.6] |
| 14 | 57 | 115 (18.3) | 107 (23.3) | 7 | 8.07 | -47.77 | 63.91 | [0.51; 15.6] |

M (SD) = Mean and standard deviation; P = Person; FB = Fitbit; MAPE = Mean absolute percentage error.

*3. Energy expenditure*

|  | Descriptive | | | | Bland-Altman | | | |
| --- | --- | --- | --- | --- | --- | --- | --- | --- |
|  | N | M (SD) CM | M (SD) FB | MAPE | Bias | Lower | Upper | 95% CI |
| Average | 58 | 2.97 (.65) | 3.31 (.81) | -11.4 | -.034 | -1.82 | 1.14 | [-.54; .14] |
| Group |  |  |  |  |  |  |  |  |
| 1 | 58 | .99 (.28) | .84 (.81) | 15.2 | .15 | -.45 | .76 | [.07; .24] |
| 2 | 57 | 3.41 (1.09) | 2.43 (1.28) | 28.7 | .99 | -.95 | 2.92 | [.73; 1.25] |
| 3 | 57 | 3.65 (.76) | 4.75 (1.06) | -30.1 | -1.1 | -3.13 | .94 | [-1.37; -.82] |
| 4 | 57 | 3.77 (.88) | 3.93 (1.04) | -4.2 | -.16 | -2.37 | 2.05 | [-.46; .14] |
| 5 | 56 | 2.80 (.67) | 4.24 (.98) | -51.4 | -1.44 | -3.27 | .39 | [-1.69; -1.19] |
| Activity |  |  |  |  |  |  |  |  |
| 1 | 58 | .97 (.30) | .84 (.20) | 13.4 | .14 | -.53 | .80 | [.05; .23] |
| 2 | 56 | .95 (.20) | .83 (.21) | 12.6 | .12 | -.39 | .63 | [.05; .19] |
| 3 | 56 | 1.01 (.22) | .84 (.20) | 16.8 | .17 | -.36 | .69 | [.09; .24] |
| 4 | 53 | 3.21 (.79) | 1.77 (1.06) | 44.9 | 1.44 | -.28 | 3.15 | [1.20; 1.68] |
| 5 | 50 | 3.95 (.99) | 3.26 (1.62) | 17.5 | .69 | -1.96 | 3.34 | [.30; 1.07] |
| 6 | 56 | 3.22 (.59) | 4.54 (.88) | -41.0 | -1.32 | -2.96 | .32 | [-1.55; -1.10] |
| 7 | 55 | 5.12 (1.44) | 4.34 (.96) | 15.2 | -.78 | -3.38 | 1.82 | [-1.14; -.42] |
| 8 | 53 | 3.24 (.76) | 4.58 (1.18) | -41.4 | -1.34 | -3.74 | 1.06 | [-1.68; -1.00] |
| 9 | 57 | 4.92 (1.09) | 4.19 (1.22) | 14.8 | .73 | -1.95 | 3.41 | [.37; 1.09] |
| 10 | 56 | 2.49 (.66) | 3.66 (.97) | -47.0 | -1.17 | -3.12 | .78 | [-1.44; -.90] |
| 11 | 56 | 3.51 (.83) | 4.34 (1.12) | -23.6 | -.38 | -3.03 | 1.36 | [-1.13; -.54] |
| 12 | 54 | 2.68 (.64) | 4.31 (.97) | -60.8 | -1.63 | -3.43 | .17 | [-1.88; -1.38] |
| 13 | 54 | 2.13 (.53) | 4.02 (.94) | -88.7 | -1.88 | -3.58 | -.22 | [-2.13; -1.66] |
| 14 | 57 | 5.14 (1.26) | 4.71 (1.49) | 8.4 | .43 | -2.81 | 3.67 | [-.01, .87] |

M (SD) = Mean and standard deviation ; CM = indirect calorimeter ; FB = Fitbit ; MAPE = Mean absolute percentage error.

*4. METs*

|  | Descriptive | | | | Bland-Altman | | | |
| --- | --- | --- | --- | --- | --- | --- | --- | --- |
|  | N | M (SD) CM | M (SD) FB | MAPE | Bias | Lower | Upper | 95% CI |
| Average | 58 | 4.73 (1.02) | 4.17 (.86) | 11.8 | .55 | -1.85 | 2.97 | [.23; .88] |
| Group |  |  |  |  |  |  |  |  |
| 1 | 58 | 1.59 (.55) | 1.06 (.23) | 33.3 | .54 | -.63 | 1.70 | [.38; .69] |
| 2 | 57 | 5.41 (1.56) | 3.08 (1.49) | 43.1 | 2.34 | -.68 | 5.35 | [1.93; 2.75] |
| 3 | 57 | 5.86 (1.14) | 5.99 (1.13) | -2.2 | -.13 | -3.31 | 3.05 | [-.56; .30] |
| 4 | 57 | 5.89 (1.35) | 4.94 (1.09) | 16.1 | .95 | -2.73 | 4.63 | [.45; 1.45] |
| 5 | 56 | 4.55 (1.02) | 5.35 (1.06) | -17.6 | -.81 | -3.74 | 2.13 | [-1.21; -.41] |
| Activity |  |  |  |  |  |  |  |  |
| 1 | 58 | 1.56 (.58) | 1.06 (.24) | 32.1 | .50 | -.74 | 1.74 | [.34; .67] |
| 2 | 56 | 1.50 (.35) | 1.05 (.24) | 30 | .45 | -.37 | 1.28 | [.34; .57] |
| 3 | 56 | 1.59 (.40) | 1.06 (.27) | 33.3 | .53 | -.39 | 1.45 | [.40; .65] |
| 4 | 53 | 5.09 (1.09) | 2.25 (1.28) | 55.8 | 2.84 | .21 | 5.46 | [2.47; 3.21] |
| 5 | 50 | 6.20 (1.27) | 4.14 (1.76) | 33.2 | 2.06 | -1.65 | 5.77 | [1.52; 2.60] |
| 6 | 56 | 5.25 (1.09) | 5.76 (.95) | -9.7 | -.50 | -3.34 | 2.33 | [-.89; -0.12] |
| 7 | 55 | 6.90 (1.44) | 6.42 (1.48) | 7 | .48 | -3.55 | 4.51 | [-.08; 1.04] |
| 8 | 53 | 5.30 (1.11) | 5.80 (1.39) | -9.4 | -.51 | -3.99 | 2.86 | [-.99; -.02] |
| 9 | 57 | 7.81 (1.59) | 5.27 (1.28) | 32.5 | 2.54 | -1.91 | 6.99 | [1.94; 3.14] |
| 10 | 56 | 3.74 (.91) | 4.61 (1.06) | -23.3 | -.87 | -3.79 | 2.04 | [-1.27; -.48] |
| 11 | 56 | 5.74 (1.22) | 5.47 (1.20) | 4.7 | .27 | -3.33 | 3.86 | [-.22; .76] |
| 12 | 54 | 4.33 (.99) | 5.45 (1.09) | -25.9 | -1.12 | -3.94 | 1.71 | [-1.51; -.72] |
| 13 | 54 | 3.43 (.83) | 5.08 (1.03) | -48.1 | -1.65 | -4.17 | .88 | [-2.00; -1.30] |
| 14 | 57 | 7.92 (1.82) | 5.92 (1.62) | 25.3 | 2 | -3.03 | 7.04 | [1.32; 2.68] |

M (SD) = Mean and standard deviation ; CM = indirect calorimeter ; FB = Fitbit ; MAPE = Mean absolute percentage error.
